# Fixation probability of rare nonmutator and evolution of mutation rates

**DOI:** 10.1002/ece3.1932

**Published:** 2016-01-11

**Authors:** Ananthu James, Kavita Jain

**Affiliations:** ^1^ Theoretical Sciences UnitJawaharlal Nehru Centre for Advanced Scientific ResearchJakkur POBangalore560064India

**Keywords:** branching process, fixation probability, fixation time, mutation rates

## Abstract

Although mutations drive the evolutionary process, the rates at which the mutations occur are themselves subject to evolutionary forces. Our purpose here is to understand the role of selection and random genetic drift in the evolution of mutation rates, and we address this question in asexual populations at mutation‐selection equilibrium neglecting selective sweeps. Using a multitype branching process, we calculate the fixation probability of a rare nonmutator in a large asexual population of mutators and find that a nonmutator is more likely to fix when the deleterious mutation rate of the mutator population is high. Compensatory mutations in the mutator population are found to decrease the fixation probability of a nonmutator when the selection coefficient is large. But, surprisingly, the fixation probability changes nonmonotonically with increasing compensatory mutation rate when the selection is mild. Using these results for the fixation probability and a drift‐barrier argument, we find a novel relationship between the mutation rates and the population size. We also discuss the time to fix the nonmutator in an adapted population of asexual mutators, and compare our results with experiments.

## Introduction

Because most mutations are deleterious, the mutation rate can not be too high; in fact, in an infinitely large population, for a broad class of fitness functions, an error threshold has been shown to exist above which the deleterious effects of mutation cannot be compensated by selection (Eigen 1971; Jain and Krug 2007). The mutation rate is not zero either (Baer et al. 2007), and it has been argued that the stochastic fluctuations in a finite population limit the evolution of mutation rates below a certain level since in small enough populations, the advantage gained by lowering the mutation rate cannot compensate the effect of random genetic drift (Lynch 2010). Empirical data for organisms with widely different effective population size show a negative correlation between the deleterious mutation rate and the population size (Sung et al. 2012), and some quantitative insight into this relationship has been obtained by treating all deleterious mutations to be lethal (Lynch 2011). However, this is clearly an extreme scenario, and it is important to ask how the deleterious mutation rate evolves when mutations are only weakly deleterious.

Many theoretical and experimental investigations have also shown that in an adapting asexual population, a mutator allele causing a higher mutation rate than that of the nonmutator can get fixed [see a recent review by Raynes and Sniegowski (2014)]. As the mutators produce not only deleterious but also beneficial mutations at a higher rate than the nonmutators, the mutator allele can hitchhike to fixation with favorable mutations (Smith and Haigh 1974; Taddei et al. 1997). However, once the population has reached a high fitness level, high mutation rates are detrimental because most mutations will now be deleterious, and in such a situation, the mutation rate is expected to decrease (Liberman and Feldman 1986). Indeed, in some experiments (Tröbner and Piechocki 1984; Notley‐McRobb et al. 2002; McDonald et al. 2012; Turrientes et al. 2013; Wielgoss et al. 2013), the mutation rate of an adapted population carrying a mutator allele has been seen to decrease and the time to fixation has been measured, but a theoretical understanding of this time scale is missing.

To address the issues discussed above, we study the fate of a rare nonmutator in a large asexual population of mutators using a multitype branching process (Patwa and Wahl 2008). An important difference between the previous works on mutator hitchhiking (Taddei et al. 1997; Andre and Godelle 2006; Wylie et al. 2009; Desai and Fisher 2011) and our study is that here the mutator population is assumed to be at mutation‐selection equilibrium and is therefore not under positive selection. However, compensatory mutations that alleviate the effect of deleterious mutations are included in our model. We find that when only deleterious mutations are present, a nonmutator can get fixed with a probability that increases with the deleterious mutation rate of the mutator. Compensatory mutations in the mutator population are expected to decrease the fixation probability of the nonmutator, and we find that this intuition is indeed correct when deleterious mutations in the mutator are effectively lethal. But, surprisingly, when the deleterious mutations are mildly harmful, the fixation probability is found to initially increase and then decrease as the rate of compensatory mutations increases. Our study thus identifies the conditions under which the spread of nonmutators is suppressed in the absence of positive selection, and complements earlier works in which a mutator hitchhikes with beneficial mutations to fixation (Taddei et al. 1997; Andre and Godelle 2006; Wylie et al. 2009; Desai and Fisher 2011).

Using our results for the fixation probability and a drift‐barrier argument which states that the advantage offered by a decrease in the deleterious mutation rate is limited by random genetic drift in a finite population (Lynch 2010), we find that the deleterious mutation rate decreases with increasing population size in accordance with experimental data (Sung et al. 2012). However, unlike previous theoretical work that treats the deleterious mutations to be effectively lethal (Lynch 2011), here we consider both strongly and weakly deleterious mutations, and not only reproduce the result in Lynch (2011), but also find a new scaling law in the latter case. We also use the results for the fixation probability to find the time to lower the mutation rate in an adapted population of mutators and compare our theoretical results with recent experiments (McDonald et al. 2012; Wielgoss et al. 2013).

## Model and Methods

We consider an asexual population in which the fitness of an individual with *k* deleterious mutations is given by $W\left(k\right)={\left(1-s\right)}^{k}$, where the selection coefficient 0 < *s* < 1. A deleterious mutation is allowed to occur at a rate $U_{d}$ and a beneficial one at a rate $U_{b}<U_{d}$. We are interested in the fate of a nonmutator that arises in this population and whose total mutation rate is smaller than that of the mutator. In a sufficiently large population of mutators in which stochastic fluctuations due to genetic drift may be ignored, this can be addressed using a branching process (Patwa and Wahl 2008), as described below.

**Figure 1:**
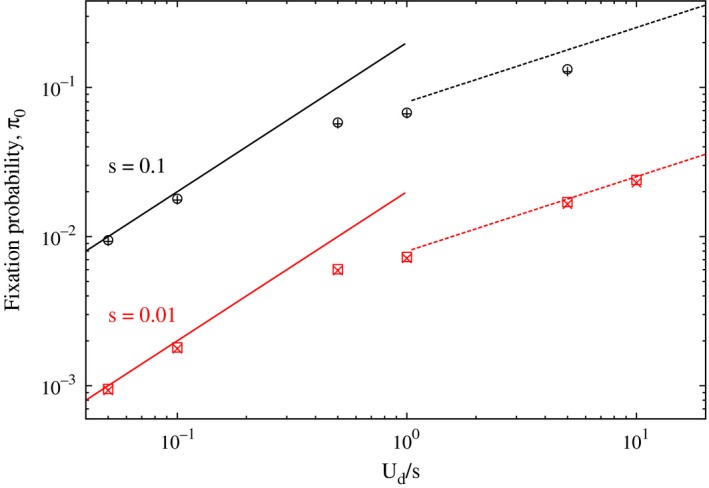
Dependence of the fixation probability obtained using a multitype branching process on the deleterious mutation rate $U_{d}$ for two values of the selection coefficient *s* and compensatory mutation rate $U_{b}=0$. The points are obtained by numerically solving (2) when the mutation rate of the nonmutator is zero (○,□), and the stationary state solution of (1) when the nonmutator's mutation rate is 50 times lower than that of the mutator (+, ×). The lines show the analytical result (6).

The fixation probability *π*(*k*,* t*) of a single copy of a nonmutator allele with fitness *W*(*k*) present at generation *t* changes according to (Johnson and Barton 2002)

(1) $$1-\pi \left(k,t\right)=\exp\left[-\frac{W(k)}{\bar{W}\left(t\right)}\sum_{k\prime }M\left(k\to k\prime \right)\pi \left(k\prime ,t+1\right)\right],$$

where $\bar{W}\left(t\right)=\sum_{k=0}^{\infty }W\left(k\right)p\left(k,t\right)$ is the average fitness of the mutator population and *p*(*k*,* t*) is the mutator frequency. The above equation expresses the fact that a single copy of the rare allele in the fitness class *k* whose offspring distribution is Poisson with mean *W*(*k*)/*W*(*t*) will be lost eventually if each of its offspring, which may undergo mutations with probability *M*(*k*→*k*′), do not survive. Here we consider strong mutators whose mutation rate is much higher than that of the nonmutator (Sniegowski et al. 1997; Oliver et al. 2000) and therefore neglect the mutation rate of the latter in most of the following discussion (however, see Fig. 1). We also assume that the mutator population is at mutation‐selection equilibrium as is likely to be the case in large populations that have been evolving for a long time in a constant environment. As a result, the probability *π*(*k*,* t*) becomes time‐independent. These considerations lead to a relatively simpler, but still highly nonlinear equation given by

(2) $$1-\pi \left(k\right)=\exp\left[-\frac{W(k)\pi (k)}{\bar{W}}\right].$$

The above expression, of course, reduces to the well‐known single locus equation (Fisher 1922; Haldane 1927) when the nonmutator can be present in only one genetic background, but here we are dealing with a multitype branching process because a nonmutator can arise in any fitness class.

The total fixation probability is obtained on summing over all genetic backgrounds (Johnson and Barton 2002),

(3) $$\pi_{\mathrm{tot}}=\sum_{k=0}^{\infty }p\left(k\right)\pi \left(k\right),$$

where the probability that a nonmutator arises in a background of *k* deleterious mutations is given by the mutator frequency *p*(*k*) in the stationary state.

Although the steady‐state frequency *p*(*k*) in the absence of compensatory mutations that mitigate the effect of deleterious mutations is known exactly (Kimura and Maruyama 1966; Haigh 1978), the corresponding solution with nonzero $U_{b}$ is not known. We therefore compute the mutator frequency numerically for nonzero $U_{b}$ using (A1) given in Appendix 1, and use these results in (2) to find the fixation probability for arbitrary $U_{b}$. To make analytical progress, we use a perturbation theory in which the effect of the small dimensionless parameter $U_{b}/s$ can be studied by expanding the quantities of interest in a power series in $U_{b}/s$, and write

(4) $$\pi \left(k\right)=\sum_{n=0}^{\infty }{\left(\frac{U_{b}}{s}\right)}^{n}\pi_{n}\left(k\right),\;p\left(k\right)=\sum_{n=0}^{\infty }{\left(\frac{U_{b}}{s}\right)}^{n}p_{n}\left(k\right).$$

The terms $\pi_{0}\left(k\right)$ and $p_{0}\left(k\right)$ corresponding to *n* = 0 in the above expansion give the results in the absence of compensatory mutations, and in Appendix 1, we calculate the stationary state fraction *p*(*k*) to linear order in $U_{b}/s$.

## Results

### Fixation probability

#### In the absence of compensatory mutations

We first consider the case when $U_{b}=0$. Taking the logarithm on both sides of (2), and expanding the left hand side (LHS) up to $\pi_{0}^{2}\left(k\right)$, we find that either $\pi_{0}\left(k\right)=0$, or

(5) $$\pi_{0}\left(k\right)=2\left(\frac{W(k)}{\bar{W_{0}}}-1\right)\approx 2s\left({\overset{¯}{k}}_{0}-k\right),$$

where the average fitness $\bar{W_{0}}=e^{-U_{d}}$ and the average number of deleterious mutations ${\overset{¯}{k}}_{0}=U_{d}/s$ (Kimura and Maruyama 1966; Haigh 1978). The last expression on the right hand side (RHS) of (5) is obtained by expanding the exponentials as the parameters $U_{d}$ and *s* are small. As the fixation probability must not be negative, the expression (5) is valid when $k<\lfloor {\overset{¯}{k}}_{0}\rfloor$, and the solution $\pi_{0}\left(k\right)=0$ holds otherwise. Here ⌊*x*⌋ denotes the largest integer less than or equal to *x*. More generally, a nonmutator can get fixed if its fitness $W\left(k\right)\approx e^{-sk}$ is larger than the average fitness $e^{-s\overset{¯}{k}}$ of the mutator population, or $k<\lfloor \overset{¯}{k}\rfloor$, $\overset{¯}{k}$ being the average number of deleterious mutations (Johnson and Barton 2002).

Equation (5) shows that the fixation probability $\pi_{0}\left(k\right)$ decreases as the number of deleterious mutations increase, as one would intuitively expect. However, the probability $p_{0}\left(k\right)$ that a nonmutator would arise in a background with $k<{\overset{¯}{k}}_{0}$ deleterious mutations increases. On summing over the backgrounds in which a nonmutator can arise, as explained in Appendix 2, we find that the total fixation probability falls in two distinct regimes defined by whether $U_{d}$ is below or above *s*:

(6) $$\pi_{0}=\sum_{k=0}^{\left\lfloor {\overset{¯}{k}}_{0}\right\rfloor }\pi_{0}\left(k\right)p_{0}\left(k\right)=\left\{\begin{array}{cc} 2U_{d}, & U_{d}\ll s\\ \sqrt{\frac{2sU_{d}}{\pi }}, & U_{d}\gg s. \end{array}\right.$$

For ${\overset{¯}{k}}_{0}\ll 1$, as a mutation is costly, it can be treated as effectively lethal (Johnson 1999). In this situation, the advantage conferred by the nonmutator is simply given by $1-e^{-U_{d}}\approx U_{d}$ and the classical result for the single locus problem gives the fixation probability to be $2U_{d}$ (Fisher 1922; Haldane 1927). For ${\overset{¯}{k}}_{0}\gg 1$, the total fixation probability apparently receives contribution from ${\overset{¯}{k}}_{0}$ genetic backgrounds, but merely $\sqrt{{\overset{¯}{k}}_{0}}$ genetic backgrounds are actually relevant because the Poisson‐distributed frequency $p_{0}\left(k\right)$ has a substantial weight for fitness classes that lie within a width $\sqrt{{\overset{¯}{k}}_{0}}$ of the mean (also, see Appendix 2). Equation (6) shows that for fixed *s*, the nonmutator is more likely to be fixed when $U_{d}$ is large. But, for a given $U_{d}$, the fixation probability initially increases with the selection coefficient and then saturates to $2U_{d}$. In Figure 1, the analytical results above are compared with those obtained by numerically iterating (2) and (1) when the mutation rate of the nonmutator is zero and $U_{d}/50$, respectively, and we see a good agreement in both cases.

#### Including compensatory mutations

**Figure 2:**
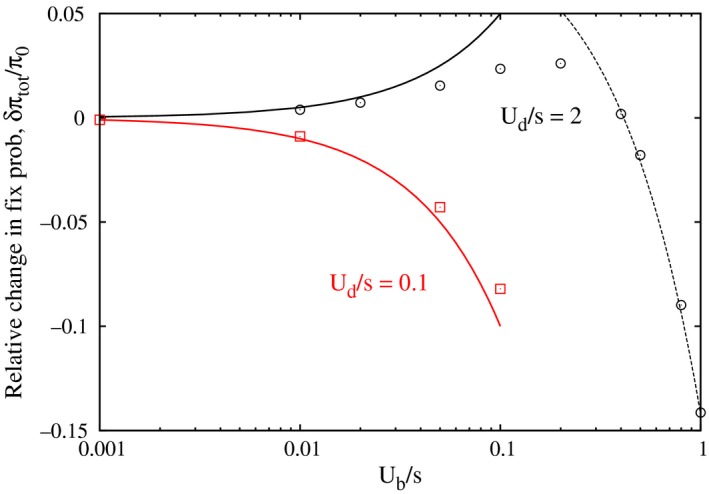
Dependence of the fixation probability obtained using a multitype branching process on the compensatory mutation rate $U_{b}$ for two values of $U_{d}/s$. The points show the numerical solution of (2), and the lines show the analytical results (11) and (12). The broken curve for $U_{b}/s>0.1$ is a linear fit, $0.1-0.24U_{b}/s$, to the numerical data. For $U_{d}/s=0.1$, the ratio $U_{b}/s$ is also below 0.1 as $U_{b}$ is assumed to be smaller than $U_{d}$.

We now study how compensatory mutations in the mutator population affect the fixation probability of the nonmutator. Figure 2 shows that when ${\overset{¯}{k}}_{0}\ll 1$, the fixation probability decreases with $U_{b}$, but for ${\overset{¯}{k}}_{0}\gg 1$, it changes *nonmonotonically*: it first increases and then decreases with increasing $U_{b}$. To understand this behavior, consider the change $\delta \pi_{\mathrm{tot}}=\pi_{\mathrm{tot}}-\pi_{0}$ in the fixation probability due to compensatory mutations which is simply given by

(7) $$\delta \pi_{\mathrm{tot}}=\sum_{k=0}^{\left\lfloor \overset{¯}{k}\right\rfloor }p_{0}\left(k\right)\delta \pi \left(k\right)+\pi_{0}\left(k\right)\delta p\left(k\right)+\delta p\left(k\right)\delta \pi \left(k\right).$$

When $U_{b}$ is nonzero, the change in the fixation probability $\delta \pi \left(k\right)=\pi \left(k\right)-\pi_{0}\left(k\right)$ and the mutator frequency $\delta p\left(k\right)=p\left(k\right)-p_{0}\left(k\right)$ behave in a qualitatively different manner. With increasing $U_{b}$, the average *fitness* of the mutator population increases which, by virtue of (2), decreases the fixation probability of the nonmutator, i.e., *δπ*(*k*) < 0. However, as the *frequency* of individuals with less deleterious mutations increases when $U_{b}$ is nonzero, the change in the mutator fraction *δp*(*k*) > 0. Thus, the change in the total fixation probability given by (7) receives both positive and negative contributions, and it is not obvious which one of these factors would have a larger effect.

To address this question, we calculate the fixation probability for small $U_{b}/s$ as described below. Substituting (4) in the expression (7) for $\delta \pi_{\mathrm{tot}}$, and neglecting terms of order ${\left(U_{b}/s\right)}^{2}$ and higher, we find that $\delta \pi_{\mathrm{tot}}\approx \left(U_{b}/s\right)\pi_{1}$, where

(8) $$\pi_{1}=\sum_{k=0}^{\left\lfloor {\overset{¯}{k}}_{0}\right\rfloor }p_{0}\left(k\right)\pi_{1}\left(k\right)+p_{1}\left(k\right)\pi_{0}\left(k\right).$$

**Figure 3:**
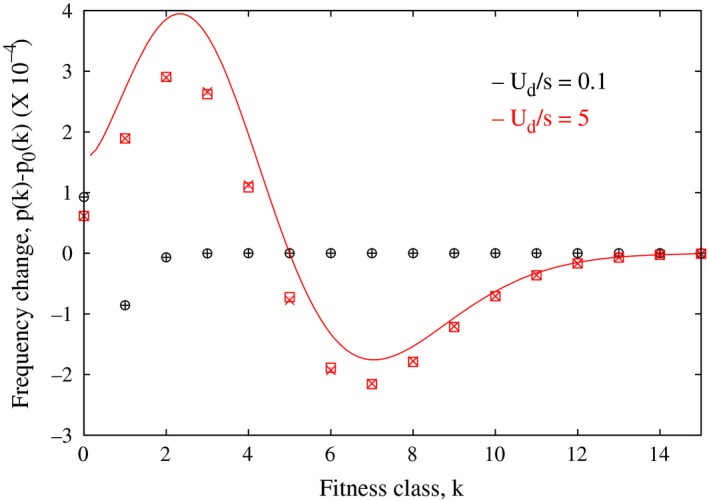
Change in the mutator frequency when compensatory mutations are included, $\delta p\left(k\right)=p\left(k\right)-p_{0}\left(k\right)$ for $U_{b}=10^{-4}$. The points (○,□) are obtained by numerically iterating (A1) and (+,×) show the perturbation theory result (A7), and we observe a good agreement. The simple expression (10) for large $U_{d}/s$ is also shown (lines).

The contribution $\pi_{1}\left(k\right)$ is calculated in Appendix 3, and we find that

(9) $$\pi_{1}\left(k\right)\approx -2s{\overset{¯}{k}}_{0}\left(1-\pi_{0}\left(k\right)\right),\;k<\left\lfloor {\overset{¯}{k}}_{0}\right\rfloor ,$$

which is negative, as expected. An expression for the fraction $p_{1}\left(k\right)$ is obtained in Appendix 3, and its behavior is shown in Figure 3 for small and large ${\overset{¯}{k}}_{0}$. For small ${\overset{¯}{k}}_{0}$, the frequency $p_{0}\left(k\right)$ is close to one in the zeroth fitness class and zero elsewhere. But the correction $p_{1}\left(k\right)$ is negligible in all the fitness classes. For large ${\overset{¯}{k}}_{0}$, the contribution $p_{1}\left(k\right)$ is significantly different from zero in many fitness classes and can be approximated by

(10) $$p_{1}\left(k\right)={\overset{¯}{k}}_{0}p_{0}\left(k\right)\ln\left(\frac{{\overset{¯}{k}}_{0}}{k}\right),k\gg 1.$$

Thus, as claimed above, the fraction $p_{1}\left(k\right)$ is positive for $k<{\overset{¯}{k}}_{0}$ and negative for $k>{\overset{¯}{k}}_{0}$ (also, see Fig. 3).

When $U_{d}\ll s$, as already mentioned, the fraction $p_{1}\left(k\right)$ is negligible in all the fitness classes and $p_{0}\left(0\right)\approx 1$. Using these results in (8) and (9), we get $\pi_{1}=-2s{\overset{¯}{k}}_{0}$, and thus

(11) $$\frac{\delta \pi_{\mathrm{tot}}}{\pi_{0}}=-\frac{U_{b}}{s},U_{b}<U_{d}<s.$$

This reduction in the fixation probability of the nonmutator when $U_{b}$ is nonzero is expected as the effect of compensatory mutation is to restore the mutators that have suffered lethal mutation to the zeroth mutation class, thus enabling them to offer competition to the nonmutators.

When $U_{d}\gg s$, as shown in Appendix 3, we can obtain a quantitative estimate of the initial increase in $\delta \pi_{\mathrm{tot}}$ by calculating the sum on the RHS of (8) to obtain (A14), and thence

(12) $$\frac{\delta \pi_{\mathrm{tot}}}{\pi_{0}}=\frac{U_{b}}{2s},U_{b}<\;s\;<U_{d}.$$

Thus, we find that for small $U_{b}$, the increase of the mutator frequency in fitness classes with fewer deleterious mutations dominates the increase in the mutator fitness resulting in positive $\delta \pi_{\mathrm{tot}}$. However, for large $U_{b}$, the net change in the fixation probability is negative because the last term in the summand of (7), which is also negative, enters the picture. As the maximum in $\delta \pi_{\mathrm{tot}}$ occurs at large $U_{b}/s$, the perturbation theory described here can not capture the eventual decrease in this parameter regime. A quantitative comparison of the results obtained by numerically solving (2) and (A1) for arbitrary $U_{b}$ with the analytical results (11) and (12) for small $U_{b}/s$ is shown in Figure 2, and we observe a good match when $U_{b}/s$ is small. For large $U_{b}/s$ and $U_{d}/s$, a fit to the numerical data shows that the fixation probability decreases linearly with $U_{b}$.

### Evolution of mutation rates in finite populations

The drift‐barrier hypothesis states that in a finite population, the beneficial effect of lower deleterious mutation rate can be outweighed by the stochastic effects of random genetic drift which limits the evolution of mutation rates (Lynch 2010). In a finite population of size *N*, a mutation that decreases the deleterious mutation rate confers an indirect selective advantage and will spread through the population. However, as $U_{d}$ decreases, the fixation probability of such a mutant decreases until it reaches its neutral value $\pi_{\mathrm{neu}}=1/N$. Here we have calculated the fixation probability $\pi_{0}$ neglecting stochastic fluctuations. The full fixation probability Π that includes the neutral and the large population limit may be obtained as follows.

The fixation time for a mutator in a finite population of nonmutators when all mutations are deleterious has been calculated using a diffusion theory by Jain and Nagar (2013), and shown to increase exponentially with the population size. The fixation probability $∼e^{-2NS}$ is thus exponentially small in the population size (Kimura 1980; Assaf and Mobilia 2011), where we have identified the rate of decrease of fixation probability with a selection coefficient 2*S*. This effective selection coefficient is found to match exactly with the result (6) for the fixation probability $\pi_{0}$ obtained here using a branching process. Although this is not a rigorous proof, these observations strongly suggest that the fixation probability of a nonmutator in a finite population of size *N* is of the classical form (Kimura 1962)

(13) $$\Pi =\frac{1-e^{-2S}}{1-e^{-2NS}},$$

where $S=\pi_{0}/2$. We also mention that the probability 2*S* depends on the difference in the deleterious mutation rate of the mutator and the nonmutator when the mutation rate of the nonmutator is nonzero (Jain and Nagar 2013), and has also been shown to be insensitive to the distribution of selective effects (Desai and Fisher 2011).

Thus, according to (13), a crossover between positive selection and neutral regime occurs when $\pi_{0}∼N^{-1}$ and gives a lower bound on the mutation rates. We recall that the fixation probability $\pi_{0}$ in (6) shows a transition when $U_{d}∼s$, and at this mutation rate, the fixation probability $\pi_{0}∼s$. This translates into a change in the behavior of $U_{d}$ when *Ns* crosses one, and we have

(14) $$U_{d}∼\left\{\begin{array}{c} {\left(sN^{2}\right)}^{-1},Ns\ll 1\\ N^{-1},Ns\gg 1. \end{array}\right.$$

**Figure 4:**
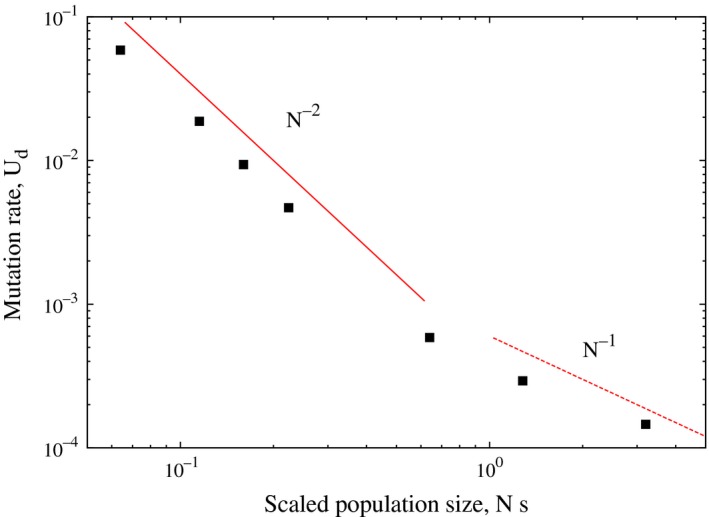
Relationship between the deleterious mutation rate and the population size for selection coefficient *s* = 0.0064 when compensatory mutations are absent. The points are obtained by numerical simulations of a Wright‐Fisher process, and the lines show the *N*‐dependence in (14).

Thus, in the weak selection regime (*Ns* ≪ 1), the deleterious mutation rate depends on the selection coefficient and decreases faster than when the selection is strong. Figure 4 shows the preliminary results of our numerical simulations for a finite size population of mutators with mutation rate $U_{d}$ in which nonmutators with mutation rate $U_{d}/2$ can arise with a certain probability. This population of nonmutators and mutators evolves via standard Wright‐Fisher dynamics, and the time to fix the nonmutators is measured (Jain and Nagar 2013). For a fixed *N*, the fixation time is found to increase as the mutation rate of the mutator is decreased until a minimum mutation rate is reached below which the fixation time remains constant. This lower bound, shown in Figure 4, exhibits different scaling behavior in the weak and strong selection regimes, in accordance with (14).

## Discussion

### Fixation probability

A rare mutator arising in a population of nonmutators carries a higher load of deleterious mutations but offers indirect benefit by producing more beneficial mutations. The fixation probability of a rare mutator in a finite nonmutator population has been studied by Andre and Godelle (2006) and Wylie et al. (2009) analytically, and found to vary nonmonotonically with the mutation rate of the mutator. It has been shown that the fixation probability is of the classical form (13) where the effective selection coefficient *S* when scaled by the selective advantage *s* increases (decreases) when the ratio of mutation rate to selection coefficient is below (above) one. Here, we studied a situation in which a nonmutator appears in a mutator population and is beneficial as it produces fewer deleterious mutations, and calculated its fixation probability $\pi_{\mathrm{tot}}$ using a branching process. The mutator population is assumed to be at mutation‐selection balance, and therefore, by definition, selective sweeps resulting in the spread of favorable mutations are neglected. However, it is interesting to note that the scaled fixation probability of the nonmutator obtained here also changes its behavior when the deleterious mutation rate is of the order of the selection coefficient, see (6). Our work significantly extends the previous result of Lynch (2011) as the deleterious effect of mutations is allowed to be mild here, and therefore, we are dealing with a truly multilocus problem.

Compensatory mutations that alleviate the effect of deleterious mutations are found to have a surprising effect on the fixation probability of the nonmutator. Although they improve the fitness of the mutator population, it also means that the nonmutator can arise in a better genetic background where it has a better chance of fixation. Thus, compensatory mutations affect both the resident mutator population and the invading nonmutator allele in a positive manner. The effect of these two factors on the fixation probability of the nonmutator is, however, opposite and can result in an unexpected increase in the fixation probability of the nonmutator when compensatory mutations are present. Here we have shown analytically that this scenario is realized when the mutations are weakly deleterious and the compensatory mutation rate is small, as illustrated in Figure 2. The increase in the fixation probability due to compensatory mutations can be quite high, but we do not have analytical estimates for this. An exact solution of (A1) would, of course, pave the way for a better analytical understanding but is currently not available.

### Fixation time

In a maladapted asexual population, the mutators can sweep the population as they facilitate rapid adaptation (Raynes and Sniegowski 2014). But as the population adapts and the supply of beneficial mutations diminishes, mutators have a detrimental effect on the population fitness and a mutation that lowers the mutation rate is favored. In bacteria *Escherichia coli*, several genes (such as *mut T* and *mut Y*) are involved in avoiding or repairing the errors that occur during the replication process, and defects in these genes can lead to the mutator phenotype (Miller 1996). But compensatory mutations in the defective error‐repair machinery can reduce the mutation rate, at least, partially (Wielgoss et al. 2013). We therefore model this situation by assigning a probability *b* with which mutators can convert into nonmutators due to a mutation in the proofreading or error‐repair region. In *E. coli*, the conversion probability *f* from nonmutator to mutators has been estimated to be $∼10^{-6}$ per bacterium per generation (Boe et al. 2000). But the probability *b* for the reverse mutation is not known, although one expects *b* < *f*, possibly because it is a gain‐of‐function mutation(Wielgoss et al. 2013).

When the rate *Nb* at which the nonmutators are produced from the mutators is small enough that the new alleles behave independently, the time taken to fix the nonmutator population is given by $T={\left(Nb\pi_{\mathrm{tot}}\right)}^{-1}$. In a long‐term evolution experiment on *E. coli*, Wielgoss et al. (2013) found the mutation rate to decrease by about a factor two in a nearly adapted mutator population with a mutation rate 150 times that of the wild type in two lineages. As the population size in Lenski's experiments has been estimated to be about $10^{7}$ (Wahl et al. 2002), the product *Nb* can be at most ten which is not too large. We first note that in the experiment of Wielgoss et al. (2013), the fixation time was longer in the lineage in which the mutation rate decreased by a smaller amount, in accordance with (6). To make a quantitative comparison, we consider the ratio of the times for the two lineages, as *T* depends strongly on the probability *b* which is not known experimentally. Using the data in Table 2 of Wielgoss et al. (2013), we find the ratio of fixation time in *mutT mutY‐L* background to that in *mutT mutY‐E* background to be 9209/5157≈1.8. The theoretical formula (6), on replacing $U_{d}$ by the difference between the mutation rate of the nonmutator and mutator, yields 1.5 (1.2) when mutations are assumed to be strongly (weakly) deleterious and the selection coefficient same in both lineages. As (6) is obtained assuming that the mutators are strong whereas the mutation rates decreased merely by a factor two in the experiment, a more careful examination is needed. Solving (1) numerically in the stationary state, we find that the ratio is unaffected when the mutations are strongly deleterious. But using the mutation rates in Table 2 of Wielgoss et al. (2013) and *s*∼0.01 yield the ratio to be about 4.5. Although the theoretical conclusions (1.5 − 4.5) are in reasonable agreement with experiments, the above analysis suggests that the reversion probability *b* may not be too small (i.e., $Nb\overset{}{\underset{∼}{>}}1$), and a more sophisticated theory that takes care of the interference between the nonmutators (Gerrish and Lenski 1998) may be required to obtain a closer match. We close this discussion by noting that in an experiment on *Saccharomyces cerevisiae* in which the adapted population reduced its genomewide mutation rate by almost a factor four in two of the experimental lines (McDonald et al. 2012), the fixation time seems to increase with the mutation rate, in contradiction with the experiment of Wielgoss et al. (2013) and the theory presented here.

### Evolution of mutation rates

Experiments show that the mutation rate decays as $N^{-0.7}$ for prokaryotes and $N^{-0.9}$ for eukaryotes (Sung et al. 2012). The population size and deleterious mutation rates are negatively correlated as deleterious mutations can get fixed in small populations due to stochastic fluctuations, but not in large populations where the genetic drift is ineffective (Lynch 2010). Here, we have shown that a reciprocal relationship between the population size and mutation rate holds for large populations, but for small populations, the deleterious mutation rate decreases much faster, see Figure 4. This is in contrast to experimental results mentioned above where the data has been fitted assuming a *single* scaling law. In view of our theoretical results discussed above, a more careful analysis of experimental data is required.

While the evolution of deleterious mutation rate has received much attention, to the best of our knowledge, analogous theoretical predictions for the beneficial mutation rate are not available. As large populations experience clonal interference (Gerrish and Lenski 1998) which results in the wastage of beneficial mutations, the rate of beneficial mutations is observed to be smaller in large populations in microbial experiments (Perfeito et al. 2007). An understanding of the relationship between the population size and the rate of beneficial mutations would be an interesting avenue to explore. Other potential factors that can affect the correlation between the mutation rate and the population size include epistasis and recombination. Here, we have also ignored the cost of fidelity, and it remains to be seen how the results presented here are affected on including it (Kimura 1967; Kondrashov 1995; Dawson 1998). A more detailed understanding of the mutation rates, both empirically and theoretically, remains a goal for the future.

## Conflict of Interest

None declared.

## Appendix 1 Mutator frequency when compensatory mutations are included

For small selection coefficient and mutation rates, the mutator frequency *p* (*k*,* t*) obeys the following continuous time equations:

(A1) $$\begin{array}{cc} \frac{\partial p(0,t)}{\partial t} & =-U_{d}p\left(0,t\right)+U_{b}p\left(1,t\right)+s\overset{¯}{k}p\left(0,t\right)\\ \frac{\partial p(k,t)}{\partial t} & =-Up\left(k,t\right)+U_{d}p\left(k-1,t\right)\\ & +U_{b}p\left(k+1,t\right)-s\left(k-\overset{¯}{k}\left(t\right)\right)p\left(k,t\right), \end{array}$$

where $U=U_{d}+U_{b}$ and $\overset{¯}{k}\left(t\right)=\sum_{k=0}^{\infty }kp\left(k,t\right)$. In the stationary state, the LHS is zero and the frequencies are time‐independent. On dividing both sides of the above equations by *s*, we find that the stationary frequency *p*(*k*) depends on the ratios $U_{b}/s$ and $U_{d}/s$. We first expand the fraction *p*(*k*) in a power series about $U_{b}/s=0$ as

(A2) $$p\left(k\right)=\sum_{n=0}^{\infty }{\left(\frac{U_{b}}{s}\right)}^{n}p_{n}\left(k\right),$$

where $p_{n}\left(k\right)$ is proportional to the *n*th derivative of *p*(*k*) with respect to $U_{b}/s$ evaluated at $U_{b}=0$. The lowest order term $p_{0}\left(k\right)$ is the solution of the steady state of (A1) in the absence of compensatory mutations, and is known to be a Poisson distribution with mean ${\overset{¯}{k}}_{0}=U_{d}/s$ (Haigh 1978):

(A3) $$p_{0}\left(k\right)=e^{-{\overset{¯}{k}}_{0}}\frac{{\overset{¯}{k}}_{0}^{k}}{k!},k=0,1,\ldots$$

To find the solution with nonzero $U_{b}$, we first set the LHS of (A1) equal to zero and substitute (A1) in these equations. On neglecting the terms of order ${\left(U_{b}/s\right)}^{2}$ and higher, we obtain the following equations for $p_{1}\left(k\right)$:

(A4) $${\overset{¯}{k}}_{1}p_{0}\left(0\right)=-p_{0}\left(1\right)$$

(A5) $$\begin{array}{c} {\overset{¯}{k}}_{0}p_{1}\left(k-1\right)-kp_{1}\left(k\right)+{\overset{¯}{k}}_{1}p_{0}\left(k\right)\\ =p_{0}\left(k\right)-p_{0}\left(k+1\right),k=1,2,\ldots \end{array}$$

where ${\overset{¯}{k}}_{1}=\sum_{k=0}^{\infty }kp_{1}\left(k\right)$. Equation (18) above immediately yields ${\overset{¯}{k}}_{1}=-{\overset{¯}{k}}_{0}$. Thus, as expected, the effect of compensatory mutations is to decrease the deleterious mutations in a population. Using this result in (A5), after some simple algebra, we get the following one‐term recursion equation for $p_{1}\left(k\right),k\geq 1$:

(A6) $$p_{1}\left(k\right)=\frac{{\overset{¯}{k}}_{0}}{k}p_{1}\left(k-1\right)-\left(\frac{1}{k}+\frac{{\overset{¯}{k}}_{0}}{k+1}\right)p_{0}\left(k\right),$$

which can be iterated easily to give

(A7) $$p_{1}\left(k\right)=\frac{{\overset{¯}{k}}_{0}^{k}}{k!}p_{1}\left(0\right)-p_{0}\left(k\right)\left[{\overset{¯}{k}}_{0}\left(H_{k+1}-1\right)+H_{k}\right],$$

where the harmonic number $H_{k}=\sum_{i=1}^{k}i^{-1}$ and the fraction $p_{1}\left(0\right)$ is determined using the normalization condition, viz. $\sum_{k=0}^{\infty }p\left(k\right)=1$. As the fraction $p_{0}\left(k\right)$ already satisfies this condition, we have the constraint $\sum_{k=0}^{\infty }p_{1}\left(k\right)=0$, on using which, $p_{1}\left(0\right)$ can be found. For large *k*, using $H_{k}\approx \ln k$ in (A7), we obtain the expression (10).

## Appendix 2 Fixation probability in the absence of compensatory mutations

To find the total fixation probability given by (3), we use the expression (5) for the fixation probability and (A3) for the mutator fraction $p_{0}\left(k\right)$ which is a Poisson distribution with mean ${\overset{¯}{k}}_{0}$. When ${\overset{¯}{k}}_{0}\ll 1$, we have $\pi_{0}\approx p_{0}\left(0\right)\pi_{0}\left(0\right)=2U_{d}$. But for ${\overset{¯}{k}}_{0}\gg 1$, on summing over the mutator backgrounds in which a nonmutator can arise, we obtain the total fixation probability to be

(A8) $$\pi_{0}=\sum_{k=0}^{\left\lfloor {\overset{¯}{k}}_{0}\right\rfloor }p_{0}\left(k\right)\pi_{0}\left(k\right)=2s\frac{e^{-\lfloor {\overset{¯}{k}}_{0}\rfloor }{\left\lfloor {\overset{¯}{k}}_{0}\right\rfloor }^{\lfloor {\overset{¯}{k}}_{0}\rfloor +1}}{\lfloor {\overset{¯}{k}}_{0}\rfloor !}.$$

On using the Stirling's formula $x!\approx \sqrt{2\pi x}{\left(x/e\right)}^{x}$ for large *x* in the last expression, we immediately obtain (6). Another way of seeing the result in the large ${\overset{¯}{k}}_{0}$ regime is by approximating the Poisson‐distributed $p_{0}\left(k\right)$ by a Gaussian with mean and variance equal to ${\overset{¯}{k}}_{0}$ and thus obtain

(A9) $$\begin{array}{cc} \pi_{0} & ∼2s\int_{{\overset{¯}{k}}_{0}-\sqrt{{\overset{¯}{k}}_{0}}}^{{\overset{¯}{k}}_{0}}dk\left({\overset{¯}{k}}_{0}-k\right)\frac{1}{\sqrt{{\overset{¯}{k}}_{0}}}e^{-\frac{{\left(k-{\overset{¯}{k}}_{0}\right)}^{2}}{2{\overset{¯}{k}}_{0}}}\\ & ∼2s\sqrt{{\overset{¯}{k}}_{0}}\int_{0}^{1}dx\;xe^{-x^{2}} \end{array},$$

where we have used the fact that the mutator frequency is substantial in the fitness classes lying within a distance $\sqrt{{\overset{¯}{k}}_{0}}$ of the mean.

## Appendix 3 Fixation probability when compensatory mutations are included

Inserting $\pi_{\mathrm{tot}}=\pi_{0}+\left(U_{b}/s\right)\pi_{1}$ and $\bar{W}={\bar{W}}_{0}+\left(U_{b}/s\right){\bar{W}}_{1}$ in (2), and using the exact equation for $\pi_{0}\left(k\right)$, we get a rather involved expression for $\pi_{1}\left(k\right)$ given by

(A10) $$\pi_{1}\left(k\right)=-\frac{{\bar{W}}_{1}}{{\bar{W}}_{0}}\frac{W\left(k\right)\pi_{0}\left(k\right)\left(1-\pi_{0}\left(k\right)\right)}{{\bar{W}}_{0}-W\left(k\right)\left(1-\pi_{0}\left(k\right)\right)}.$$

As all the parameters are smaller than one, we work with the approximate expression (5) for the probability $\pi_{0}\left(k\right)$ and arrive at (9).

We now calculate the contribution $\pi_{1}$ given by (8) when ${\overset{¯}{k}}_{0}\gg 1$ using the expression (9) for $\pi_{1}\left(k\right)$ and the frequency $p_{1}\left(k\right)$ in (10). We have

(A11) $$\pi_{1}=-2s{\overset{¯}{k}}_{0}\left(1-\sum_{k=\lfloor {\overset{¯}{k}}_{0}\rfloor }^{\infty }p_{0}\left(k\right)\right)+2s\sum_{k=0}^{\infty }\left({\overset{¯}{k}}_{0}-k\right)p_{1}\left(k\right)-\sum_{k=\lfloor {\overset{¯}{k}}_{0}\rfloor }^{\infty }p_{1}\left(k\right)\pi_{0}\left(k\right)$$

(A12) $$=2s{\overset{¯}{k}}_{0}\sum_{k=\lfloor {\overset{¯}{k}}_{0}\rfloor }^{\infty }p_{0}\left(k\right)-2\sum_{k=\lfloor {\overset{¯}{k}}_{0}\rfloor }^{\infty }\left({\overset{¯}{k}}_{0}-k\right)sp_{1}\left(k\right),$$

where we have assumed that $U_{b},U_{d},s$ are small, but $U_{b}/s$ and $U_{d}/s$ are finite. The last expression is obtained on using the normalization condition $\sum_{k=0}^{\infty }p_{1}\left(k\right)=0$ and the expression for the average ${\overset{¯}{k}}_{1}$. For large ${\overset{¯}{k}}_{0}$, we approximate the Poisson distribution $p_{0}\left(k\right)$ by a Gaussian as

(A13) $$p_{0}\left(k\right)\approx \frac{1}{\sqrt{2\pi {\overset{¯}{k}}_{0}}}e^{-\frac{{\left(k-{\overset{¯}{k}}_{0}\right)}^{2}}{2{\overset{¯}{k}}_{0}}}\left(1-\frac{k-{\overset{¯}{k}}_{0}}{2{\overset{¯}{k}}_{0}}+O\left({\overset{¯}{k}}_{0}^{-2}\right)\right).$$

Approximating the sums in (A12) by integrals, we finally have

(A14) $$\frac{\pi_{1}}{s}\approx \left[{\overset{¯}{k}}_{0}-\sqrt{\frac{{\overset{¯}{k}}_{0}}{2\pi }}\right]-\frac{2{\overset{¯}{k}}_{0}}{\sqrt{\pi }}\int_{0}^{\infty }dz\frac{e^{-z^{2}}}{1+z\sqrt{\frac{2}{{\overset{¯}{k}}_{0}}}}\approx \sqrt{\frac{{\overset{¯}{k}}_{0}}{2\pi }}$$

where we have carried out an integration by parts in the second integral on the RHS and neglected subleading terms in ${\overset{¯}{k}}_{0}$.
